# Murine nasal-associated lymphoid tissue (NALT) harbors human alphaherpesvirus 1 (HSV-1) DNA during latency, and dexamethasone triggers viral replication

**DOI:** 10.1128/jvi.02251-24

**Published:** 2025-03-26

**Authors:** Kelly S. Harrison, Shannon R. Cowan, Clinton Jones

**Affiliations:** 1 Department of Veterinary Pathobiology, Oklahoma State University, College of Veterinary Medicine 70729, Stillwater, Oklahoma, USA; University of Toronto, Toronto, Ontario, Canada

**Keywords:** HSV-1, nasal-associated lymphoid tissue, quiescent/latent infection, dexamethasone

## Abstract

Human alphaherpesvirus 1 (HSV-1) acute infection causes conjunctivitis, encephalitis, genital lesions, and herpes esophagitis. Following acute infection, HSV-1 and other alpha-herpesvirinae subfamily members establish life-long latency in neurons within the trigeminal ganglia and central nervous system. Notably, certain animal alpha-herpesvirinae subfamily members, including bovine alphaherpesvirus 1 (BoHV-1), canine herpesvirus 1, equine herpesvirus 4, and pseudorabies virus, establish a quiescent/latent infection in tonsils. BoHV-1 viral gene expression and virus shedding from tonsils also occur during reactivation from latency in calves. Consequently, we tested whether nasopharyngeal lymphoid tissue (NALT) harbors HSV-1 DNA in latently infected mice because it is structurally and functionally comparable with tonsils. NALT prepared from latently infected mice consistently contained viral DNA, but infectious virus was not detected. In contrast to latently infected TG neurons, the HSV-1 latency-associated transcript was not detected in NALT of latently infected mice. HSV-1 DNA levels, immediate early RNA expression, and virus shedding were readily detected when NALT explants were cultured with a medium containing the synthetic corticosteroid dexamethasone for 48 h. Increased viral DNA and virus production were not detected in NALT explants when incubated with a medium lacking dexamethasone. Sorting cells from NALT of HSV-1 latently infected mice revealed that dendritic cells, microfold cells, and natural killer cells, but not B or T cells, harbor HSV-1 DNA, and infectious virus was readily detected when cultured in medium containing dexamethasone. In summary, certain NALT cells consistently contain viral DNA in latently infected mice, and dexamethasone triggers viral gene expression and virus production.

**IMPORTANCE:**

Human alphaherpesvirus 1 (HSV-1) acute infection causes various diseases, including herpes esophagitis. HSV-1 subsequently establishes lifelong latency in neurons within the trigeminal ganglia and central nervous system. Viral DNA, but not infectious virus, was consistently detected in nasopharyngeal lymphoid tissue (NALT) of latently infected mice. NALT is structurally and functionally comparable with the tonsils of other mammals, including humans. RNA and protein expression of infected cell protein 0 (ICP0) and ICP4 plus virus production were consistently detected when NALT explants were cultured with a medium containing dexamethasone, a synthetic corticosteroid. Sorting NALT cells from HSV-1 latently infected mice revealed dendritic cells, microfold cells, and natural killer cells that harbor HSV-1 DNA. Virus shedding was readily detected when viral DNA-positive NALT cells were cultured in a medium containing dexamethasone. These studies revealed that specific NALT cells harbor viral DNA, and dexamethasone triggered viral replication and virus production, suggesting that reactivation from a latent or quiescent infection had occurred.

## INTRODUCTION

Infection of craniofacial mucosal membranes with human alphaherpesvirus 1 (HSV-1) leads to life-long latency in neurons of trigeminal ganglia (TG), brainstem, and other regions of the central nervous system (1, 2). HSV-1 causes serious recurrent eye infections (3) and fatal encephalitis (4, 5). Interestingly, HSV-1 is also the second leading cause of infectious esophagitis, known as herpes esophagitis (HE), reviewed in (6). The majority of HE infections occur in immune-compromised people, for example, people infected with HIV, cancer patients undergoing chemotherapy, organ transplant patients, or patients treated with corticosteroids (6–8). HSV-1 can also cause HE in healthy people (9, 10). HE can cause inflamed vesicular lesions and ulcers along the mid-distal esophagus, which leads to difficulty in swallowing, fever, cough, and vomiting. Severe HE cases can cause upper respiratory distress, gastrointestinal bleeding, and occasionally death.

The HSV-1 latency-reactivation cycle is operationally divided into three distinct steps: establishment, maintenance, and reactivation (1, 2). During the establishment of latency, a subset of infected neurons survive, viral gene expression is silenced, and latency is established. Maintenance of latency also requires survival of infected neurons, restricted lytic cycle viral gene expression, and little or no virus shedding. Fever, UV light, and increased cortisol triggered by stress can initiate reactivation of latency, reviewed in (11).

**Fig 1:**
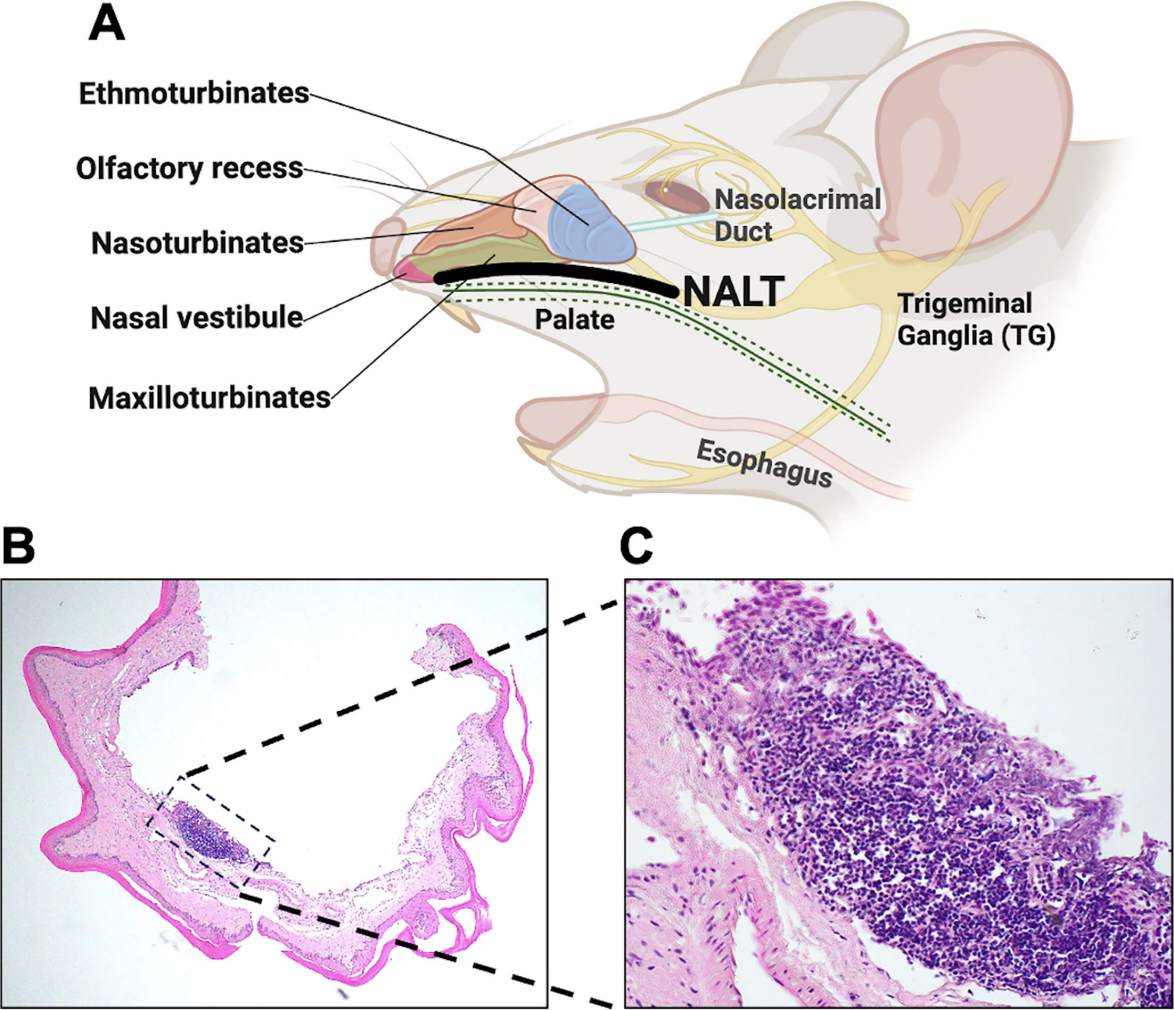
Schematic of mouse oropharyngeal anatomy. (A) Image showing nasopharyngeal-associated lymph tissue (NALT) located anterior to the soft palate, along the floor of naso-, maxillo-, and ethmo-turbinates. Fluid from the ocular cavity drains via nasolacrimal ducts into nasal and oral cavities. Image created with BioRender.com. (B) H&E staining showing sagittal sections of isolated soft palate (4× magnification). (C) Dashed lines highlight the location of NALT that was magnified (40× magnification).

Although neurons are the primary site for latency, HSV-1 was detected in palatine tonsils (PT) of a subset of people who had a tonsillectomy or adenectomy due to chronic lymphoid hyperplasia (12). No evidence of acute HSV-1 infections was observed in these people. As expected, Epstein-Barr Virus DNA, but not HSV-2 or human cytomegalovirus DNA, is also detected in PT of these people. Notably, viral DNA from other α-herpesvirinae subfamily members, bovine alphaherpesvirus 1 (BoHV-1) (13, 14), pseudorabies virus (15), and canine herpesvirus 1 (16) is consistently detected in pharyngeal tonsil and other lymphoid tissue during latency of their respective natural hosts. Within 30 min after latently infected calves are given an intravenous injection of the synthetic corticosteroid dexamethasone (DEX), infected cell protein 4 (ICP4) RNA is the only viral gene expressed in pharyngeal tonsil (17). At 3 and 6 h after DEX treatment, all lytic cycle viral transcripts are readily detected, indicating reactivation from latency occurs rapidly in pharyngeal tonsil and not indirectly via TG neurons. *In situ* hybridization studies revealed that a higher number of pharyngeal tonsil cells express BoHV-1 lytic viral transcripts as a function of time after DEX treatment, confirming that the viral gene expression occurs in pharyngeal tonsils (14). Pharyngeal tonsils, also referred to as adenoids and lingual tonsils, are present in humans and other mammals (18). Rodents, including mice, lack tonsils; however, they contain nasal-associated lymphoid tissue, also referred to as nasopharynx-associated lymphoid tissue (NALT) (18). NALT is proposed to have similar functions as human palatine and nasopharyngeal tonsils (19). NALT is localized near the front of the soft palate (Fig. 1A and B) where it forms a non-encapsulated lymphoid aggregate comprised of T cells, B cells, high endothelial venules (HEVs), dendritic cells (DCs) covered by epithelium with rare goblet cells, and microfold cells (Fig. 1C).

This study revealed that certain cells within NALT of latently infected mice harbor viral DNA, but not infectious viruses. Incubating NALT cells with media containing DEX significantly increased the expression of key immediate early viral transcriptional regulators (ICP0 and ICP4), viral DNA levels, and infectious viruses. Dendritic cells (DC), microfold (M) cells, and natural killer (NK) cells, but not B or T cells, contain viral DNA in NALT of mice latently infected with HSV-1. Based on these studies, we suggest that HSV-1 contributes to HE or other respiratory tract complications.

## RESULTS

### Detection of HSV-1 in NALT of latently infected mice

**Fig 2:**
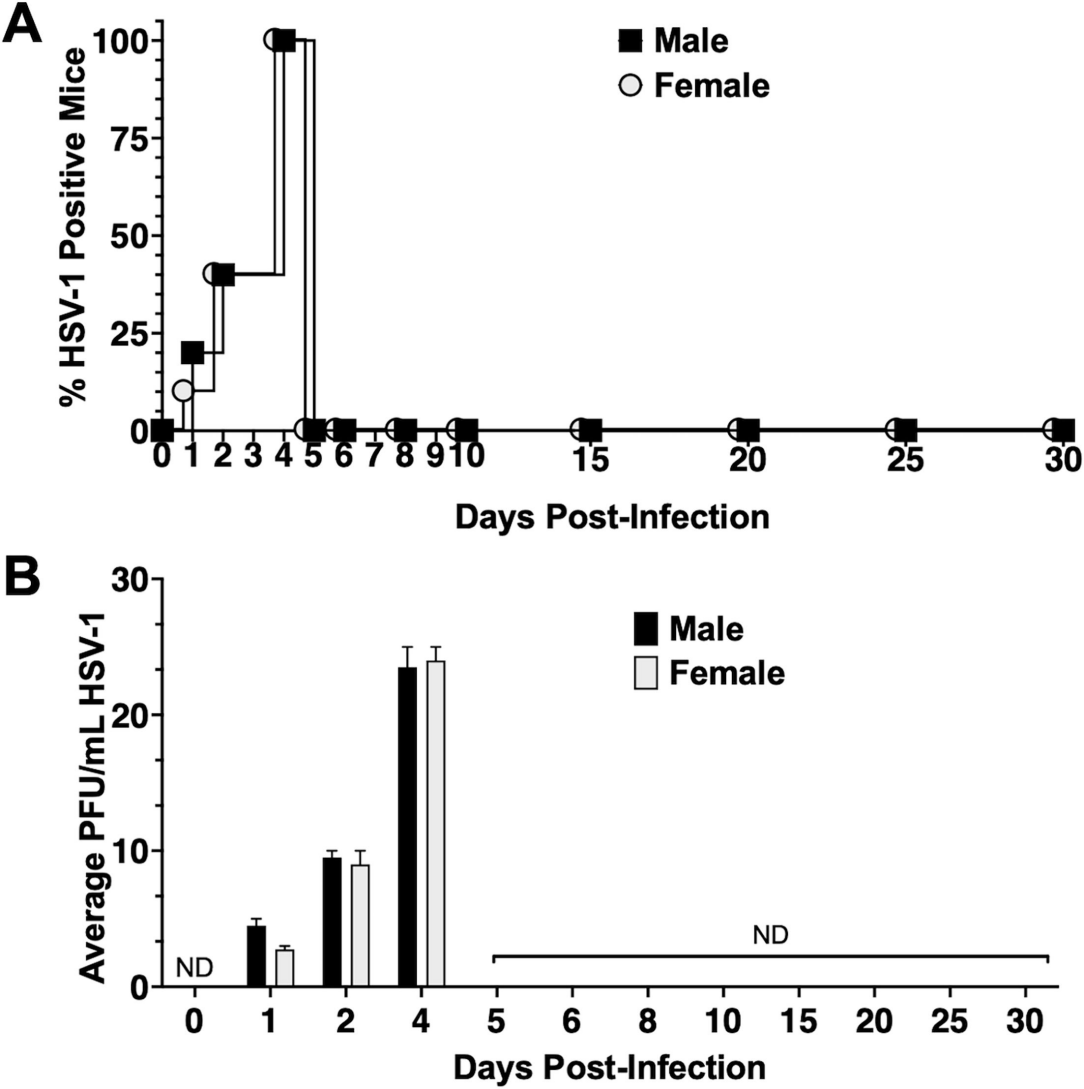
Oral swabs following ocular infection with HSV-1. Nine-week-old male and female C57Bl/6 J mice (*n* = 5) were ocularly infected with 10^5^ PFU HSV-1 strain McKrae per eye without scarification. Day 0 is ocular swabs from uninfected mice. Oral swabs were collected to test for infectious virus as follows: each day from day 1 through day 5 post-infection, every other day for days 5–10 post-infection, and every 5 days for days 10–30 post-infection. Data are shown as (A) percent HSV-1 positive animals/sex and (B) mean + SEM plaque-forming unit (PFU) per mL for males and females at each time point. ND: none detected.

Initial studies tested whether HSV-1 was present in NALT of latently infected mice following ocular infection. Nine-week-old male and female C57BL/6 J mice (*n* = 4 or 5) were ocularly infected with 10^5^ PFU HSV-1 strain McKrae without scarification as described previously (20, 21). After HSV-1 infection, oral swabs were collected daily for the first 5 days, every other day from days 6 to 10, and every 5 days from days 10 to 30. Approximately 25% of males had detectable HSV-1 in oral swabs 1 day post-infection (dpi) (Fig. 2A) with an average titer of only five plaque-forming units (PFU) per mL (Fig. 2B). By 2 dpi, males and females had equal virus titers (approximately 10 PFU/mL), and by 4 dpi, all mice had detectable HSV-1 in oral swabs with approximately 25 PFU/mL. After 5 dpi, HSV-1 was not detected in oral swabs. Swabs from the ocular cavity or oral swabs were not detected after latently infected mice were euthanized (data not shown). These studies demonstrated that following ocular infection, low levels of infectious HSV-1 virus were detected in the oral cavity.

**Fig 3:**
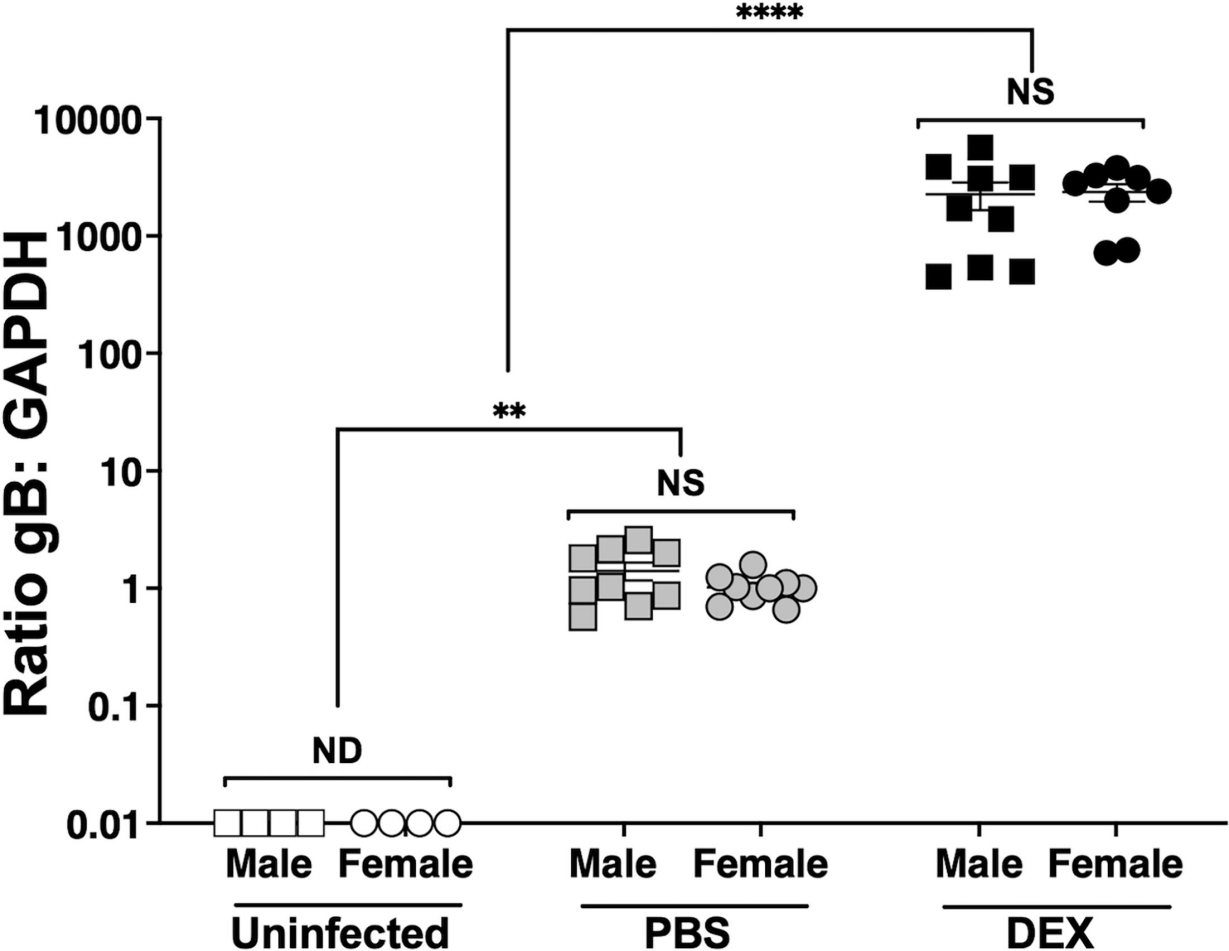
Detection of HSV-1 DNA in NALT explants prepared from latently infected mice. 30 days post-infection (latency), NALT were isolated (*n* = 4–5 mice, two separate experiments) and explanted in either MEM plus PBS or 10 µM DEX for 48 h. Uninfected mice were included as controls. DNA was prepared and used for quantitative PCR using primers for HSV-1 glycoprotein B (gB) and housekeeping control gene GAPDH. Data are shown as individual data points with mean + SEM; ND: None detected; NS: Not significant; **P < 0.005; ****P < 0.0001 using Student’s *t*-test.

Thirty days post-infection, mice are operationally defined as latently infected because the infectious virus is not detected from the cornea, conjunctiva, or TG (20, 21). NALT (Fig. 1B and C) was prepared (*n* = 4–5 mice/group, two independent experiments) and subsequently explanted in MEM containing 2% charcoal-stripped fetal bovine serum (FBS) and 10 µM DEX or PBS as a negative control. DEX accelerates HSV-1 reactivation in TG explants (20, 22), suggesting that this treatment induces viral replication and shedding. NALT from uninfected animals was included as controls. The rationale for using stripped FBS for these studies is that lipid-based molecules including hormones, certain growth factors, and cytokines are removed. However, this process does not remove salts, glucose, and most amino acids. Forty-eight hours post-explant, DNA was prepared from explanted NALT cultures, and quantitative PCR (qPCR) was performed using primers specific for the HSV-1 envelope glycoprotein B (gB) and a housekeeping gene, glyceraldehyde 3-phosphate dehydrogenase (GAPDH). Viral DNA from NALT of uninfected mice was undetectable (Fig. 3, white symbols). Latently infected NALT explanted in PBS for 48 h had low levels of HSV-1 DNA (Fig. 3, gray symbols) that were significantly higher than uninfected mice. In sharp contrast, NALT explanted in MEM, 2% stripped FBS, and DEX for 48 h contained significantly higher levels of gB DNA copies compared with NALT from latently infected male or female mice (Fig. 3, black symbols). These studies revealed NALT explants cultured in MEM plus DEX for 48 h contained significantly higher HSV-1 DNA levels when compared with NALT prepared from latently infected mice that contained MEM and PBS, indicating viral DNA replication occurred.

### Infectious HSV-1 was detected in NALT explanted in MEM containing DEX

**Fig 4:**
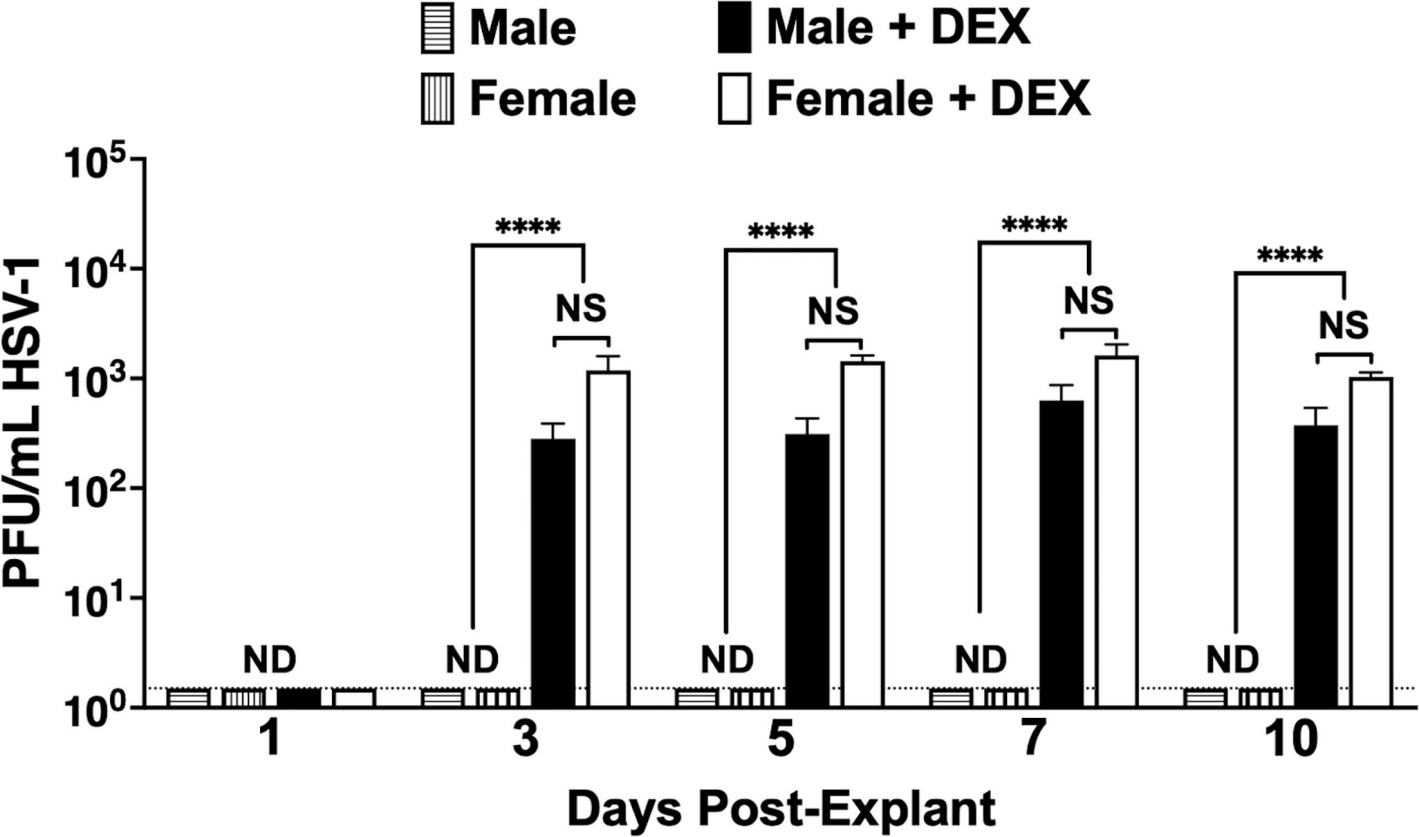
Incubation of explanted NALT with MEM containing DEX triggers virus production. At 30 dpi (latency), NALT from male and female mice (*n* = 5, two experiments) latently infected with HSV-1 were collected and minced into smaller pieces. NALT explants were incubated with MEM, 2% charcoal-stripped FBS, and 10 µM DEX or PBS. Each day, 500 µL of aliquots was collected, and plaque assays were performed to measure the infectious virus. Data are shown as mean + SEM; dashed line indicates the limit of detection for HSV-1. ND: None detected; NS: Not significant; ****P < 0.0001 using Student’s *t*-test.

Subsequent studies tested whether infectious virus levels in NALT increased after explant. NALT prepared from latently infected mice was incubated with MEM and DEX or PBS as described in Materials and Methods. Aliquots (~500 µL) of cell-free supernatant were collected every other day for 10 days post-explant (dpe). Infectious virus was not detected one dpe regardless of DEX treatment (Fig. 4). At three dpe, infectious virus was detected in male and female NALT explants if incubated with DEX (Fig. 4), which is similar to previous results in TG (20, 21). The infectious virus was also detected in male and female NALT explants if treated with DEX at 5, 7, and 10 dpe. Approximately 500 PFU/mL was produced for males and 1000 PFU/mL for females; however, these differences were not significantly different. NALT explants from uninfected mice incubated with MEM + 2% stripped FBS and PBS did not produce detectable levels of infectious virus (data not shown). In summary, NALT explants from latently infected mice produced an infectious virus when incubated with MEM that contained DEX, which was consistent with increased viral DNA levels (Fig. 3).

### HSV-1 gene expression in NALT during latency and reactivation

**Fig 5:**
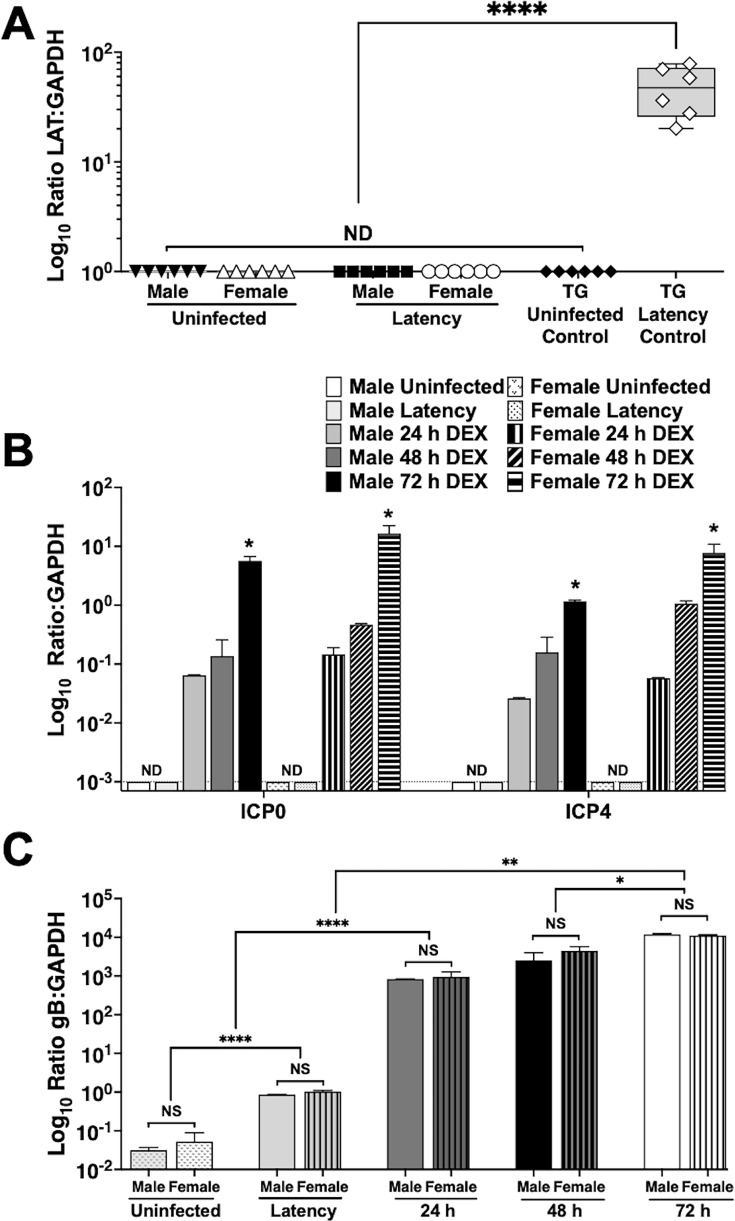
HSV-1 gene expression in NALT during latency and DEX-induced reactivation. (A) NALT from male and female uninfected mice or mice latently infected with HSV-1 (*n* = 3, two separate experiments) were isolated in Trizol; RNA was purified, and RT-qPCR was performed using primers for HSV-1 LAT and mouse GAPDH. TG were included as UI-negative and LAT-positive controls. Data are shown as box plots from max to min values with a line at the median. (B and C) NALT from male (solid) and female (pattern) uninfected or latently infected mice (*n* = 4 mice, two separate experiments) was explanted in MEM + 2% stripped FBS with 10 µM DEX. At the indicated times, tissues and supernatant were collected, RNA isolated, and RT-qPCR was performed using primers for HSV-1 ICP0, ICP4 (B), gB (C), and mouse GAPDH. Data are shown as mean + SD. ND: None detected; dotted line: limit of detection. *P < 0.05; **P < 0.005; ****P < 0.0001 using Student’s *t*-test.

LAT is the only viral RNA that is abundantly expressed in TG during latency, reviewed in (1, 2). To investigate whether LAT is expressed in NALT of latently infected mice, tissue from uninfected and latently infected mice was prepared, and total RNA was prepared for quantitative reverse transcriptase-PCR (RT-qPCR) (Fig. 5A). As expected, LAT expression was readily detected in TG from latently infected mice, and LAT:GAPDH ratios were approximately 100. However, LAT was not detected in NALT prepared from mice latently infected with HSV-1. LAT was not detected in TG or NALT (Fig. 5A; black diamonds) prepared from uninfected mice.

To evaluate the expression of key lytic cycle viral genes, RT-qPCR was performed using primers that specifically amplify infected cell protein 0 (ICP0) or ICP4 sequences. ICP0 protein is encoded by an IE transcript that activates viral gene expression (23) and is detected during the early stages of HSV-1 reactivation from latency (20). ICP4 is an IE transcriptional regulatory protein that binds double-stranded DNA and recruits RNA polymerase II to facilitate the transition from immediate early gene expression to early and late gene expression (24). ICP0 and ICP4 RNA expressions were below the limits of detection (Fig. 5B, dotted line) in RNA prepared from NALT of uninfected mice or latently infected mice (Fig. 5B). Conversely, when NALT was incubated with MEM and 10 µM DEX, ICP0 and ICP4 RNA expressions increased temporally in mice regardless of sex when compared with GAPDH RNA levels. By 72 hpe, ICP0 RNA expression was approximately 100-fold higher than GAPDH. ICP4 RNA expression increased significantly by 72 hpe for male and female NALT when incubated with 10 µM DEX. Approximately 50-fold higher levels of ICP4 RNA levels were detected compared with GAPDH levels in males and a 125-fold increase in females.

Finally, RT-qPCR was used to compare gB RNA expression when NALT was explanted in the presence of DEX (Fig. 5C). Consistent with ICP0 and ICP4 RNA expressions, gB RNA expression increased over time, but there were no significant differences between male and female mice. As expected, gB RNA expression in NALT from latently infected mice was significantly higher than in uninfected mice but less than NALT incubated with MEM and DEX for 24, 48, and 72 h. In summary, these studies revealed DEX significantly increased ICP0, ICP4, and gB RNA levels in NALT explants.

### ICP0 and ICP4 protein expression occurred in NALT explants when incubated with MEM containing DEX

**Fig 6:**
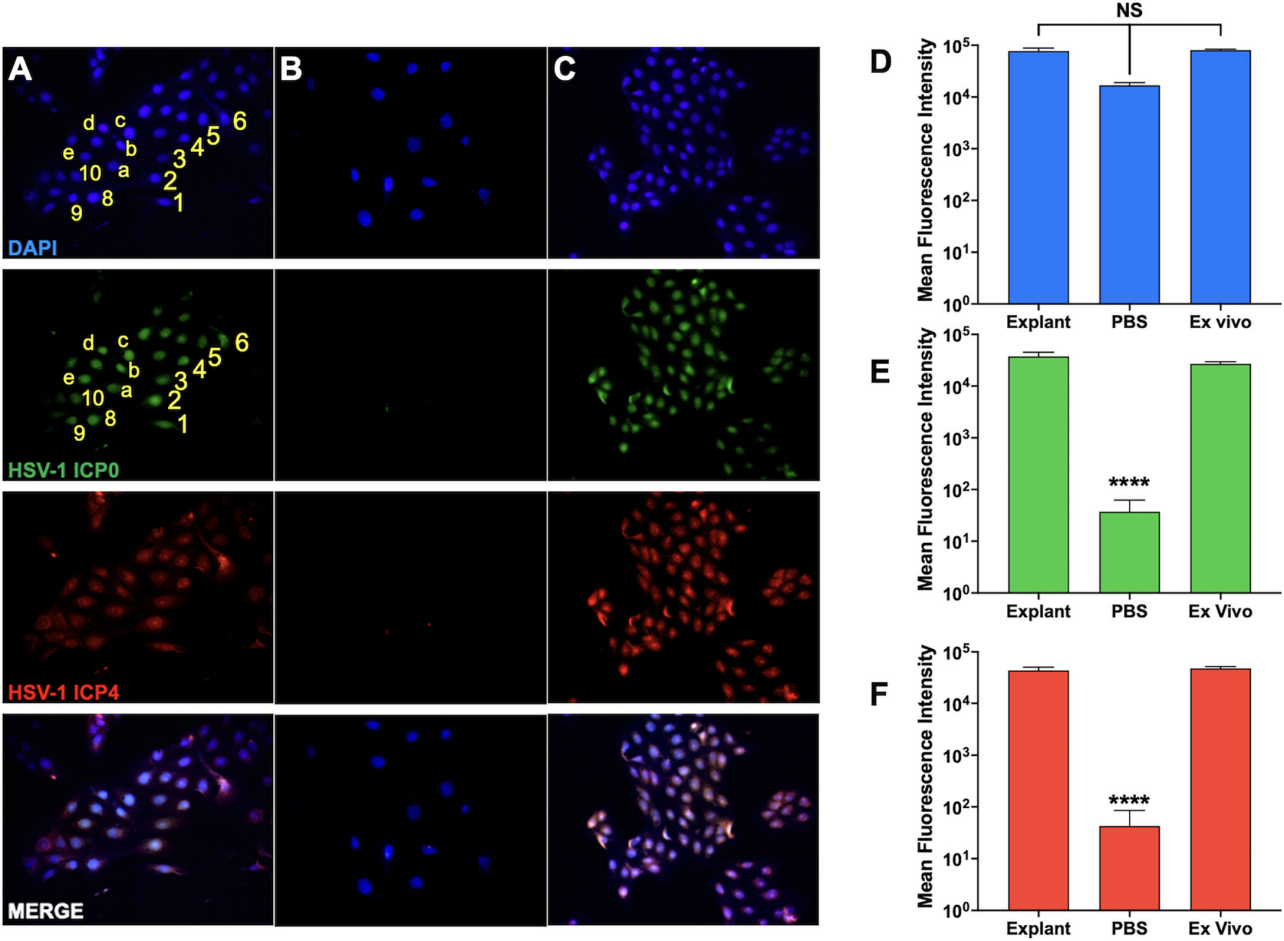
Immunofluorescence of NALT single cells. Single-cell suspensions were prepared from NALT of HSV-1 latently infected mice (*n* = 5). These cells were incubated in MEM + 2% stripped FBS and either 10 µM DEX (A) or PBS (B) for 48 h. The cells from NALT of uninfected mice were also collected and then infected ex vivo with HSV-1 using a multiplicity of infection of 1 for 48 h (C). The cells were then fixed and stained for HSV-1 ICP0 (green), ICP4 (red), and DAPI (blue) to identify cells that expressed the viral proteins (merge). Representative images were captured at 60× magnification with autoexposure using a BioTek Cytation 5 Cell imager and Gen5 software. (D-F) Relative fluorescence was calculated using ImageJ for (D) DAPI stained nuclei, (E) HSV-1 ICP0, and (F) HSV-1 ICP4. Data are shown as mean + SEM fluorescence intensity calculated from an average of 500 cells per field of view; NS: Not significant; ****P < 0.0001 using one-way ANOVA with multiple comparison post-test.

Studies described above used minced NALT from palate and NALT for the detection of viral DNA, RNA, and infectious virus following explant. The following studies were performed using single-cell suspensions prepared from NALT of latently infected mice. Cell suspensions were cultured in MEM + 2% stripped FBS and 10 µM DEX for 48 h to induce virus production. The cells were fixed and stained for ICP0 (Fig. 6; green) or ICP4 (red). A subset of the total ICP0+ and DAPI+ cells are denoted 1–10 and a-e. Of 41 cells, 26 of these cells were DAPI+ and ICP0+. Furthermore, these results revealed that all ICP0+ cells were DAPI+ indicating ICP0 was primarily localized in the nucleus. In summary, these studies revealed that ICP0 and ICP4 were localized in the nucleus of cells prepared from male (Fig. 6A) and female mice (data not shown) when cultured with 10 µM DEX at similar levels of fluorescence (Fig. 6E and F).

When cell suspensions were incubated with MEM + 2% stripped FBS + PBS for 48 h (no DEX treatment), ICP0 and ICP4 protein expressions were not detected (Fig. 6B). Furthermore, the mean fluorescence intensity was significantly higher in male and female cells when incubated with MEM that contained DEX when compared with cells incubated with MEM and PBS (Fig. 6E and F). When NALT single cells were prepared from uninfected animals and then infected (Fig. 6C), equal levels of ICP0 and ICP4 expressions were observed in “ex vivo” infected cells incubated with DEX (Fig. 6E and F). In summary, these studies demonstrated NALT cells infected with HSV-1 lead to ICP0 and ICP4 expressions, and both viral proteins were detected in the nucleus.

### Fluorescence-activated cell sorting (FACS) of NALT cell subtypes

In addition to epithelial cells, NALT consists of B and T cells, dendritic cells (DCs), and specialized microfold cells expressing intermediate levels of CD45 (CD45^int+^), which differentiates them from CD45^−^, cytokeratin 18-expressing epithelial cells (25–27). Single-cell suspensions were generated from NALT of latently infected mice, and antibodies directed against key cell subtypes (Table 1) were used to fluorescently stain and sort the denoted cell populations. Since dramatic sex-specific differences were not observed in the studies described above, only female mice were used for testing whether sorted NALT cells contain viral DNA.

**TABLE 1:** Cell markers of the respective cell types, fluorochrome, and antibody dilution factor

Marker	Primary cell type	Fluorophore/filter and dilution
Live/Dead	All	Propidium iodide (PE-Texas Red filter); 1:500
CD45	Pan-leukocyte	PerCP; 1:100
CD3	T cells	FITC; 1:100
CD20	B cells	APC; 1:100
CD11c	Dendritic cells	Dylight 405; 1:100
NK1.1	Natural killer cells	Pacific Blue; 1:100
Cytokeratin 18	Microfold cells	PE; 1:100
NeuN	Neurons	Alexa-488; 1:1,000

**Fig 7:**
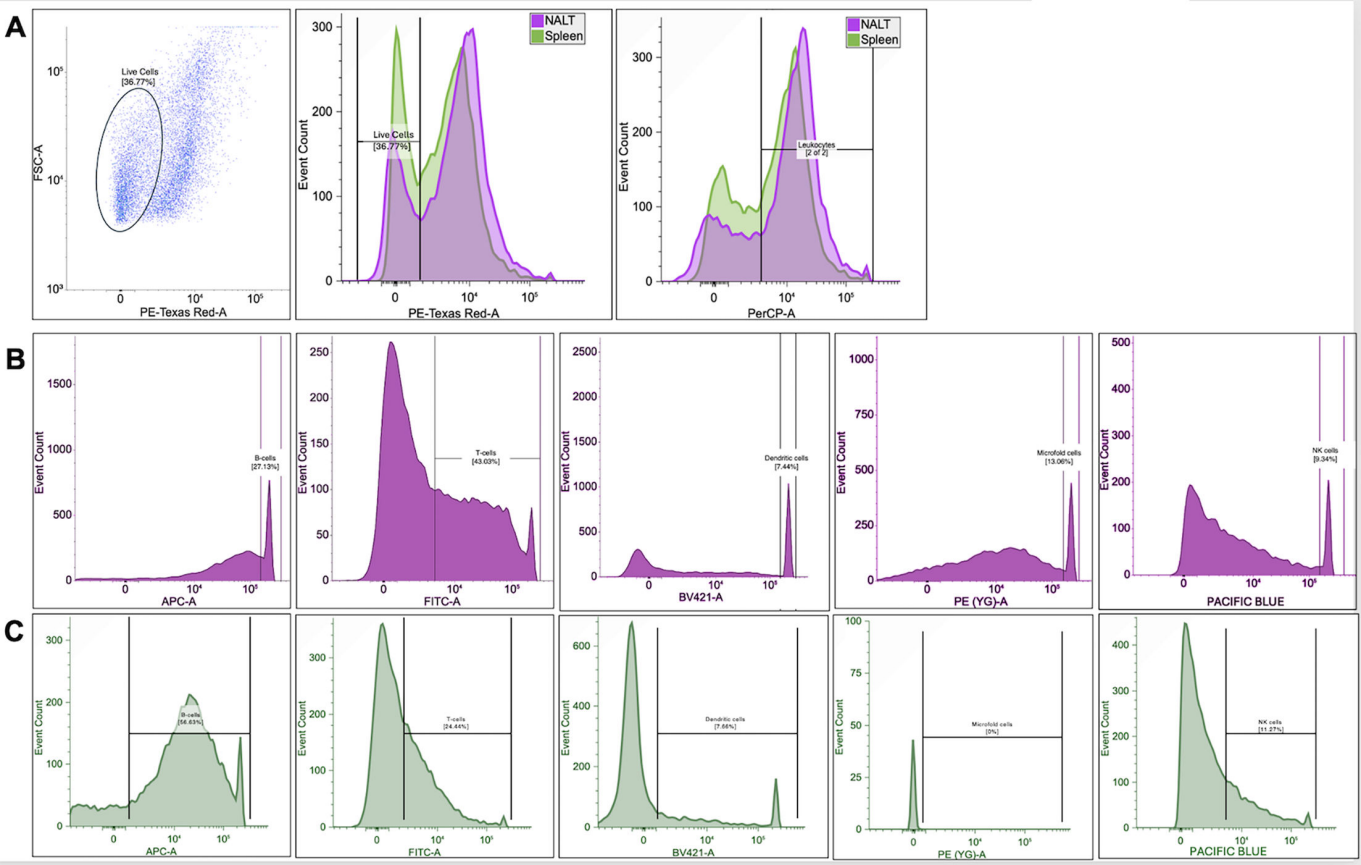
FACS and gating strategy of NALT single-cell suspension. To identify which specific cell types that harbor HSV-1 DNA, single-cell suspensions were stained from HSV-1 latently infected female mice (*n* = 10, two separate experiments) for immune cell markers and sorted on a BD FACSAria. First, total leukocytes were identified from propidium live/dead stain (A). Within the leukocyte population, B cells, T cells, dendritic cells, NK cells, and M cells were sorted (B). Splenocytes were included as controls (C). Gates were conservatively set to sort for purity over yield.

Initial singlets were gated using forward scatter area (FSA) and height (FSH, data not shown). Live cells from the singlet cell populations were gated using propidium iodide (Fig. 7, panel A, left and center); approximately 37% of the total cell population was viable. Within live cells, CD45^+^ leukocytes were gated (Fig. 7, panel A, right); spleen samples were included as controls (Fig. 7, panel A green; panel C). Extreme edge-gating strategies and sorting for purity were applied to prevent cross-contamination of cell types. Total leukocyte populations were further sorted into cell subtypes; CD45^+^CD20^+^ B cells were the second highest in number at 27% (Fig. 7B). CD45^+^CD3^+^ T cells accounted for the highest percent of the gated subtypes with 43%, which resulted in 150,000 T cells and 200,000 sorted B cells. In the spleen, B cells comprised 56%, but T cells were only 24% of the total population (Fig. 7C).

CD45^+^CD11c^+^ dendritic (DC), CD45^INT+^CK18^+^ microfold (M), and CD45^+^NK1.1^+^ natural killer (NK) cells comprised the smallest populations of cells in both NALT and spleen. DC cells accounted for approximately 7.5% of gated leukocytes, whereas NK cells only accounted for 9% in NALT. M cells from NALT comprised 13% of total leukocytes but were not present in the spleen as expected. Approximately 50,000 DC, NK, and microfold cells were collected from NALT after cell sorting. Distribution of the respective cell types is consistent with independent studies (25–27).

### Identification of NALT cells that contain HSV-1 DNA in latently infected mice

**Fig 8:**
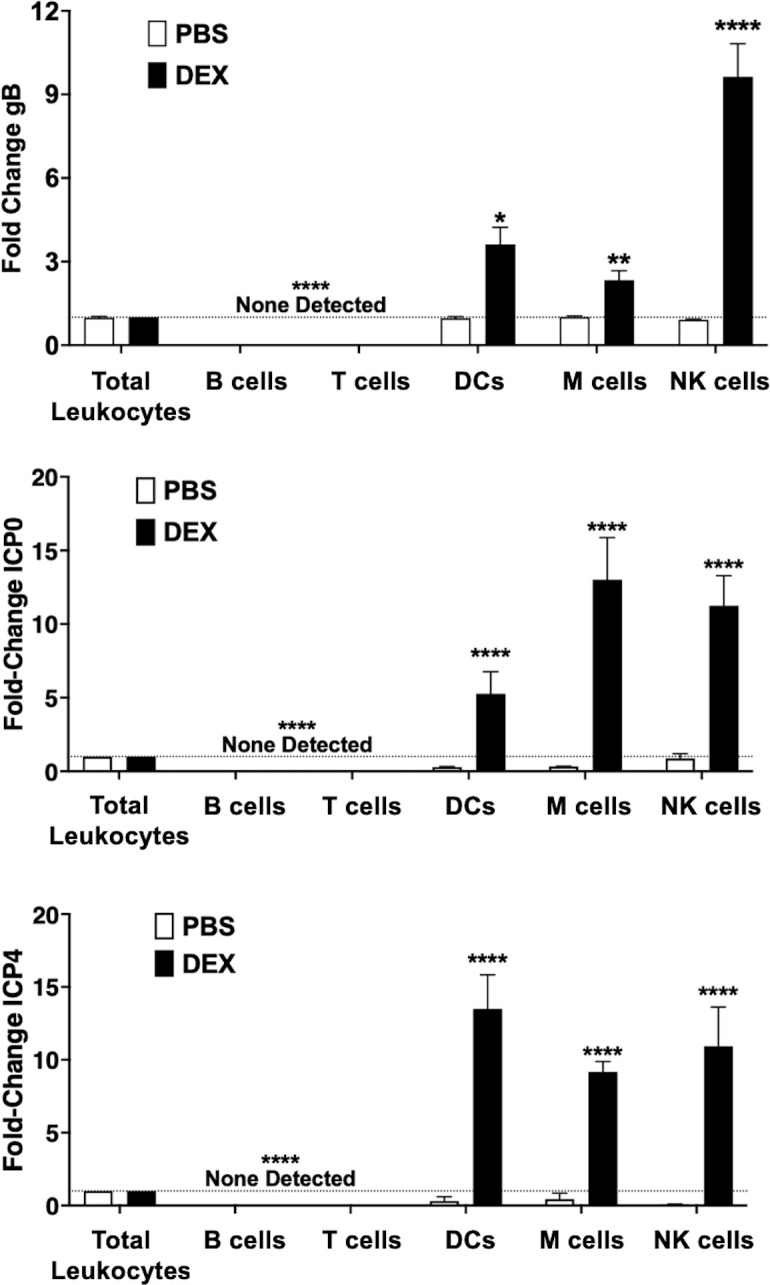
Quantitative PCR from FACS NALT to identify cells harboring HSV-1 DNA. Following FACS of single-cell suspension from HSV-1 latently infected NALT, cells were either cultured with PBS or 10 µM DEX for 24 h. ~ 10^4^ cells were used for genomic analysis: (A) DNA from cells was used for qPCR with HSV-1 gB primers and cellular GAPDH housekeeping gene. Data are shown as mean ± SEM fold change compared with total leukocytes. (B and C) RNA from each cell type was used for RT-qPCR using primers for HSV-1 ICP0 (B) or ICP4 (C). Data are shown as mean + SEM fold change relative to total leukocytes from triplicate wells. *P < 0.05, **P < 0.005, ****P < 0.0001 using one-way ANOVA with multiple comparison post-tes.

Following cell sorting of NALT prepared from female mice latently infected with HSV-1, individual cell types were cultured in MEM, 2% stripped FBS, and PBS (Fig. 8, white bars) or 10 µM DEX (Fig. 8, black bars) for 24 h to induce HSV-1 replication. Compared with whole palate containing NALT in which tissues were incubated with DEX for 48 h (Fig. 3), sorted leukocyte populations were incubated with DEX for 24 h to reduce potential cytotoxic effects of DEX on sorted immune cell subtypes. Viability studies (trypan blue exclusion assay) revealed this time point did not induce high levels of cell death in the respective sorted cell types, regardless of whether cells were from uninfected or latently infected mice (data not shown). DNA was prepared from approximately 10^4^ sorted cells per subtype, and qPCR was performed (Fig. 8A). HSV-1 gB and mouse GAPDH DNA levels were used to identify cell type(s) that contained abundant levels of HSV-1 DNA relative to gB DNA levels in total leukocytes. When sorted cells were incubated with 10 µM DEX for 24 h, gB DNA levels were 9 times higher in NK cells from female mice relative to total leukocytes (Fig. 8A, black bars). Consistent with unsorted NALT, gB DNA levels were not elevated compared with total leukocytes when cells were only incubated with MEM but no DEX (Fig. 8A, white bars). Microfold (M) cells and dendritic cells (DCs) also contained higher levels of gB DNA (approximately 3-fold and 4-fold, respectively) when cultures were incubated with 10 µM DEX. Conversely, HSV-1 DNA was not detected in B cells or T cells regardless of incubation with DEX.

RT-qPCR was subsequently used to measure ICP0 (Fig. 8B) or ICP4 RNA levels (Fig. 8C). ICP0 and ICP4 RNA levels were significantly increased in DCs, M cells, and NK cells from latently infected mice incubated with MEM containing 10 µM DEX for 24 h when compared with expression levels in total leukocytes. ICP0 RNA expression was 12-fold higher in M cells, 10-fold higher in NK cells, and 5-fold higher DC in cells. ICP4 RNA expression was 13-fold higher in DCs when latently infected mice were incubated with 10 µM DEX. M cells and NK cells contained approximately a 10-fold increase in ICP4 expression versus leukocytes. ICP0 and ICP4 RNA expressions were not detected in B or T cells from NALT of latently infected mice regardless of whether cultures were incubated with PBS or 10 µM DEX. In summary, DEX treatment of NK cells, DCs, and microfold cells contained significantly higher ICP0 RNA levels, ICP4 RNA levels, and gB DNA levels following DEX treatment. In sharp contrast, incubating sorted B and T cells with MEM containing DEX, there was no increase in viral DNA or ICPO and ICP4 RNA levels.

### Detection of infectious virus in certain sorted NALT cells after treatment with MEM containing DEX

**Fig 9:**
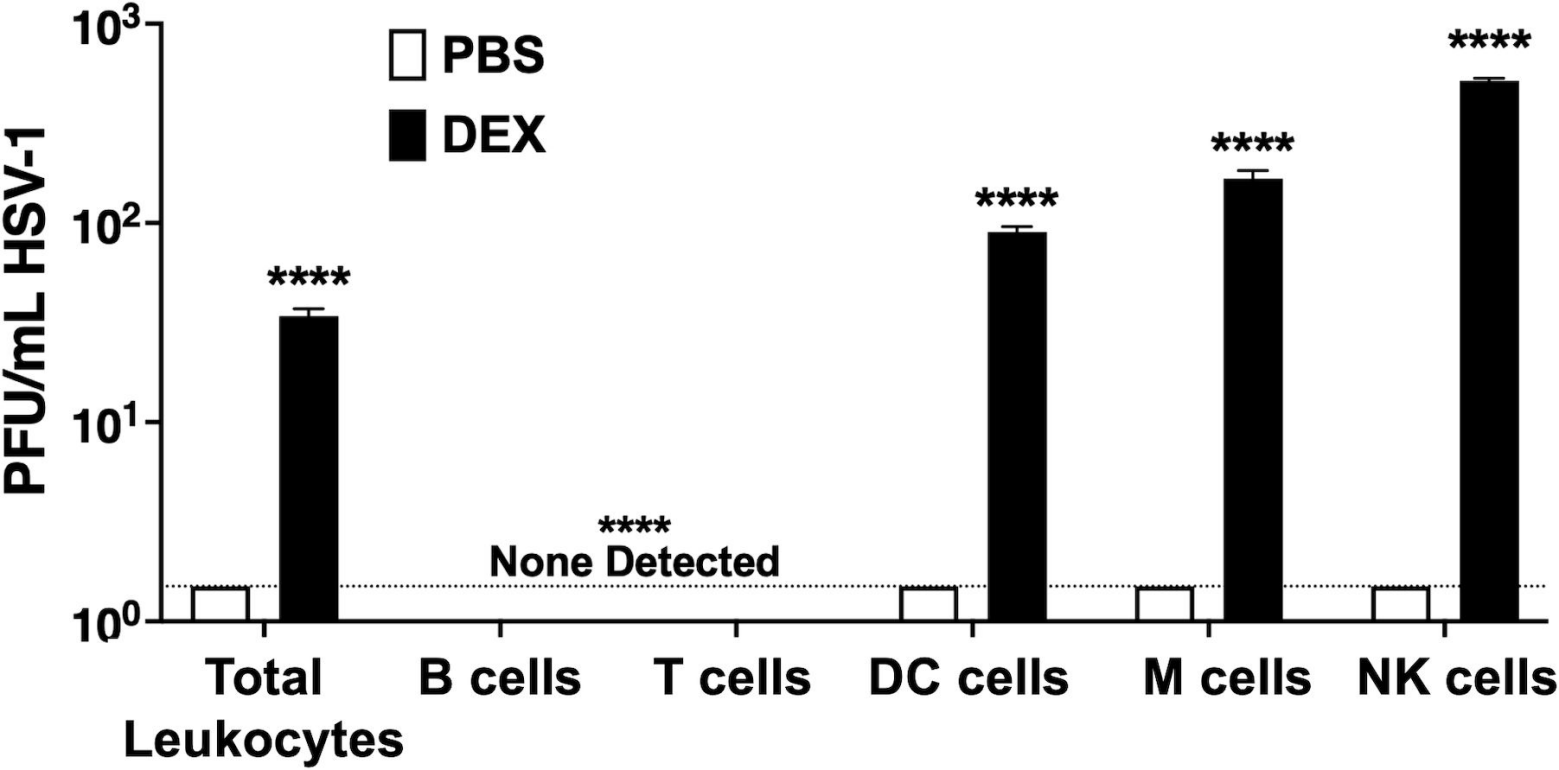
Sorted NALT cell types produced infectious virus after incubation with MEM and DEX. In total, 10^4^ cells sorted from NALT of HSV-1 latently infected female mice (*n* = 10, two separate experiments) were cultured in MEM + 2% stripped FBS and either PBS or 10 µM DEX for 24 h then used to plaque on Vero cells for infectious virus. Data are shown for mean + SEM for triplicate wells of duplicate experiments. ****P < 0.0001 using one-way ANOVA with multiple comparison post-test.

Additional studies tested whether the infectious virus was produced in sorted NALT cells treated with DEX. Approximately 10^4^ sorted cells were cultured with MEM containing 10 µM DEX or PBS for 24 h. Cells were subsequently subjected to serial freeze/thawing (−80°C to 37°C), and cell-free supernatants were prepared. Plaque assays were performed in Vero cell monolayers after incubation with the cell-free supernatants (Fig. 9). DCs cultured with DEX resulted in approximately 100 PFU/mL, which was significantly higher than cells cultured in MEM with no DEX. Approximately 150 PFU/mL of HSV-1 virus was detected in M cells, whereas NK cells generated more than 500 PFU/mL when incubated with MEM, 2% stripped FBS, and DEX (Fig. 9, black bars). Infectious HSV-1 was not detected in B or T cells cultured in MEM and DEX, which was consistent with the absence of detectable viral DNA in these cells.

### Neurons were not detected in sorted NALT cell types

**Fig 10:**
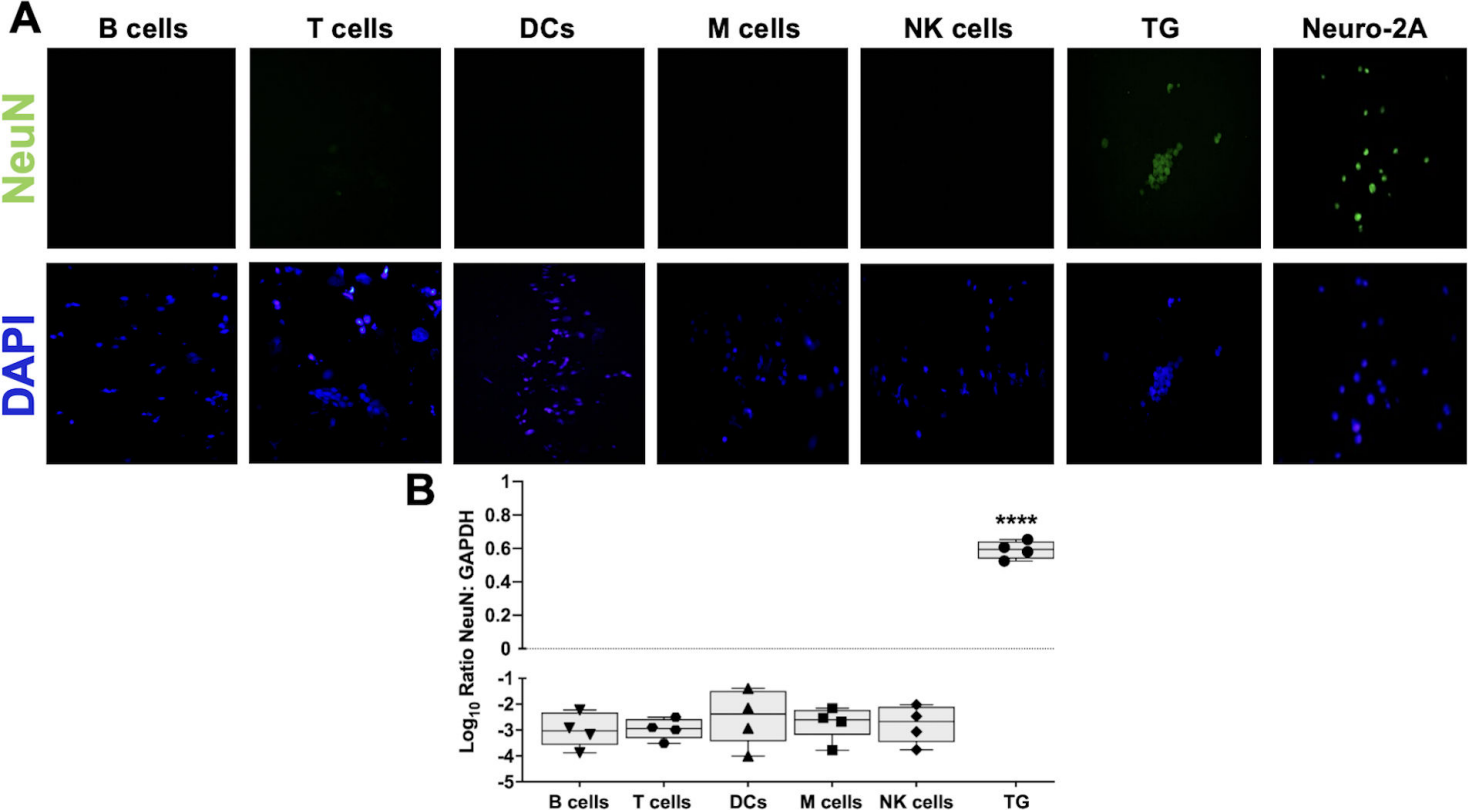
NeuN staining of NALT-sorted cell subtypes. (A) NALT cell types were sorted using the denoted antibodies in Table 1. Following selection with these antibodies, the cells were fixed and stained for the intracellular neuronal markers NeuN (green) and DAPI (blue). NeuN-positive trigeminal ganglia (TG) were sorted and included as positive controls along with the mouse neuroblastoma cell line (Neuro2A). Images were obtained at 40× magnification using an Olympus BX microscope and CellSense Entry software. Representative images from sorted T cells showing NeuN overexposure were included. All DAPI-stained samples were exposed for 3.4 ms. Exposure after NeuN antibody staining was 2.85 ms for TG, 6.3 ms for Neuro-2A cells, and 250 ms for sorted NALT cells. (B) 10,000 FACS-sorted NALT cells were collected in TRIzol, and RNA was prepared for RT-qPCR to measure NeuN expression levels. Neurons sorted from TG were included as positive controls. Data are shown as box-and-whisker plots from max to min of Log_10_ ratio NeuN to GAPDH with a line at the median; duplicate wells from two experiments. A dashed line represents the limit of detection for qPCR. ****P < 0.0001 using one-way ANOVA with multiple comparison post-test.

In mice, TG and associated craniofacial nerve networks create a complicated plexus, with the maxillary branch (V2) terminating in NALT (25, 28, 29) (Fig. 1). Like humans, the mandibular V3 division of TG contains sensory and motor neurons, and special visceral efferent (SVE) axons that extend to the palatine nerve via synaptic connections to the glossopharyngeal nerve (CN IX) and vagus nerve (CN X) (30, 31). To determine whether DEX-induced HSV-1 DNA and viral gene expression were not due to contaminating neurons present in the respective sorted NALT cell types of latently infected mice, immunocytochemistry was performed using antibodies directed against the neuron marker NeuN that is present in the nucleus (32). NeuN-positive neurons were sorted from mouse TG and included as a positive control (Fig. 10A, top row); mouse neuroblastoma cells (Neuro-2A) were also included as positive controls. NeuN staining colocalized with DAPI-stained nuclei in sorted TG neurons and undifferentiated Neuro-2A cells as previously reported (33). Despite exposing NALT cells more than 80 times longer than TG images, NeuN staining was not detected. Only when NALT cells were exposed more than 1,000 times longer than TG were we able to see background NeuN staining (data not shown). To further confirm the absence of neuron contamination, RNA from sorted cell types was prepared and used for RT-qPCR with primers specific to NeuN and GAPDH. RNA prepared from sorted TG neurons was included as a positive control (Fig. 10B, circles). NeuN RNA expression in TG neurons was approximately 4 times higher than GAPDH levels. In contrast, all sorted leukocyte subtypes had NeuN RNA expression levels relative to ratios significantly lower than TG and below the limit of detection (Fig. 10B, dotted line). NeuN RNA levels in the sorted cells were approximately 10^−3^ times lower than GAPDH RNA levels. In summary, these studies demonstrated that sorted NALT cells were not contaminated with detectable numbers of neurons.

## DISCUSSION

HE is a frequently overlooked complication of HSV-1 craniofacial infections. For example, it is not clear whether HE is due to (i) HSV-1 reactivation from latency in TG and virus spread to the throat; (ii) drainage of infectious virus from the nasolacrimal duct system during lytic infection; and/or (iii) cells in the esophageal region harboring latent viral DNA that support reactivation from latency or quiescent infection. To provide insight into these complicated virus-host interactions, a mouse ocular model of infection was tested for the presence of HSV-1 DNA in NALT of latently infected mice. These studies revealed HSV-1 established a quiescent/latent infection in NALT following ocular infection of mice. Notably, the synthetic corticosteroid DEX treatment consistently triggered viral gene expression, replication, and production of infectious virus.

HSV-1 ocular infection and replication on mucosal surfaces culminates in blinking and drainage of ocular fluids to the lacrimal canaliculi and nasolacrimal duct (34). Within the lacrimal duct, the ophthalmic nerve (V1) of the TG conjoins parasympathetic nerves from CN7 controlling the tear reflex and nasolacrimal duct function. Consequently, ocular and nasal fluid is redirected to the throat (29), suggesting that infectious HSV-1 particles are transported through this duct to the throat during acute infection. These events are consistent with the detection of HSV-1 in oral swabs until day 6 days after infection of mucosal surfaces of the cornea and conjunctiva (Fig. 2).

Incubating NALT single-cell suspensions in MEM that contains 2% stripped FBS and DEX significantly increased viral DNA (Fig. 3), production of infectious virus (Fig. 4), and expression of ICP0 and ICP4 RNA (Fig. 5) within 72 h. Furthermore, sorted DCs, M cells, and NK cells but not B or T cells prepared from NALT-induced ICP0 plus ICP4 RNA expression and virus shedding (Fig. 8 and 9) when incubated with MEM, 2% stripped FBS, and DEX within 24 h after incubation. These studies are consistent with DEX-inducing virus production during explant-induced TG reactivation from latency (20, 35). It is also possible that prolonged incubation of NALT or the respective viral DNA + sorted NALT cells may lead to virus shedding when incubated with MEM and no DEX. HSV-1 replication in cultured cells is also stimulated by DEX (35, 36), in part because the glucocorticoid receptor transactivates cis-regulatory modules crucial for activating expression of ICP0, ICP4, ICP27, and VP16 (11, 35, 37, 38). We suggest that DEX-mediated activation of the glucocorticoid receptor in NALT cells that harbor viral DNA in latently infected mice stimulates viral gene expression, which culminates in the production of infectious virus.

In contrast to HSV-1 latency in TG neurons, LAT was not readily detected in NALT or sorted cells that harbor viral DNA. Although this result was unexpected, BoHV-1 does not express the latency-related (LR) gene in tonsils of latently infected calves unless nested PCR and then southern blotting are used to detect LR-RNA (17). The LR gene (39), like LAT (1, 2), is abundantly expressed in TG neurons during latency, and these genes play important roles during the latency-reactivation cycle. Since the LAT promoter is more active in neurons, we suggest cellular transcription factors important for LAT promoter activity are not present in NALT cells that harbor HSV-1 DNA in latently infected mice. Although neurons are clearly the primary site for HSV-1 latency, previous reports concluded that HSV-1 establishes a quiescent infection in non-neuronal cells. For example, quiescent HSV-1 infections can occur in non-neuronal cultured cells, and the infectious virus subsequently produced in these cultures (40, 41). HSV-1 DNA has also been detected in tonsils from patients undergoing tonsillectomy and adenectomies (12, 42–44).

In conclusion, DC, NK, and M cells harbor HSV-1 DNA in NALT of latently infected mice and produce infectious virus following DEX treatment. M cells in NALT are important components of the mucosal immune system (45). Cytokeratin 18 is a specific marker for bovine intestinal M cells (46); however, it does not appear to be specific for M cells in human palatine tonsils (47). It is clear M cells are present in murine NALT (48); however, this study appears to be the first to use cytokeratin 18 to identify M cells in murine MALT. Since M cells were initially sorted in the leukocyte population, using the Cytokeratin 18-specific antibodies to further sort M cells generated a relatively pure population of cells. Notably, DC, NK, and M cells only comprise approximately 15% of the total lymphocyte population in NALT (Fig. 7). Future studies will focus on identifying cellular factors that maintain this latent/quiescent infection in NALT cells versus TG neurons. Although the results in this study have similarities to other animal α-herpesvirinae subfamilies, additional studies are needed to demonstrate that HSV-1 consistently establishes a quiescent or latent infection in tonsils of humans as demonstrated in the natural host of BoHV-1 (13, 14), pseudorabies virus (15), and canine herpesvirus 1 (16).

## MATERIALS AND METHODS

### Viruses and cell lines

Vero monkey kidney cells (ATCC CCL-81) and Neuro-2A (ATCC CCL-131) were grown in minimal essential medium (MEM; Corning) supplemented with 10% FBS (Atlas Biologicals), 2  mM L-glutamine (Corning), and antibiotics (100 IU/mL penicillin, 100  µg/mL Streptomycin, Cytiva Hyclone) at 37°C, 5% CO2. HSV-1 strain McKrae was obtained from the late Dr. Steven Wechsler (University of California, Irvine Medical School) and grown in Vero cells until >80% CPE (cytopathic effect) was observed. Viral aliquots were obtained through serial freeze-thawing of cells at 37°C/−80°C and subsequent centrifugation to remove cellular debris. Virus was titered on Vero cell monolayers to determine PFU/mL for each stock prior to infection of mice.

### Animal studies

C57BL/6 J mice (male and female, 6–8 weeks old) were purchased from Jackson laboratories and allowed to acclimate to standard laboratory conditions (12 h light/dark cycles, five animals/cage) for 1 week prior to ocular infection. Following acclimation, animals were anesthetized via standard isoflurane/oxygen vaporization and infected ocularly with 10^5^ PFU/mL HSV-1 strain McKrae without scarification as previously described (21, 35).

NALT preparation was performed as previously described (25) with modifications. Briefly, following humane euthanasia via isoflurane overdose, the head was removed and dissected sagittally along the midline. Tapered Ultra Fine Point forceps were inserted behind the fore teeth at the tip of the nose and used to gently peel the palate containing NALT from the remaining nasal tissue. Following palate isolation, tissues were collected directly in formalin or MEM for downstream applications.

Formalin-fixed tissues were processed with assistance from the Oklahoma Center for Respiratory Infectious Diseases Immunopathology Core (OCRID) facility. Briefly, formalin-fixed tissues were paraffin-embedded and trimmed to 5 µm, collected on positively charged slides, and then routinely stained with hematoxylin and eosin (H&E) for microscopic evaluation.

### Reverse transcription and quantitative PCR (RT/qPCR)

Soft palate containing NALT was prepared as described and cultured in MEM with or without DEX for 24 or 48 h where indicated. Kidneys were included as internal negative reference tissues as they do not contain any HSV-1 DNA following ocular infection. The use of an internal reference tissue within the same animal as experimental samples provides a 2-fold reduction in technical variation and PCR efficiency (49). Following incubation, the samples were collected and briefly spun to collect any free-floating cells, the supernatant was removed, and alkaline lysis buffer (25 mM NaOH, pH 12) was added and boiled for 1 h, as previously described (50). Lysis buffer was neutralized with 40 mM Tris-HCl pH 5 and briefly centrifuged to remove cell debris.

For individual cell suspensions, the cells were pelleted and suspended in 100 µL Diethyl pyrocarbonate (DEPC)-water freeze/thawed three times at −80°C/37°C and subsequently boiled at 99°C for 15 min to ensure cell lysis. This method was used because of the limited number of cells, preventing oversalting and sample loss.

Following cell lysis, two rounds of phenol:chloroform:isoamyl alcohol (25:24:1) extraction were performed followed by one round of chloroform: isoamyl alcohol (24:1  vol/vol) and ethanol precipitation. Two volumes of ice-cold 100% ethanol were added and incubated at −20°C overnight. DNA was pelleted via centrifugation (18,000 g for 30 min), washed twice with 100% ethanol twice, and air-dried prior to dissolving in DEPC-water. A total of 10 ng of genomic DNA was used as a template for Sybr Green qPCR (Applied Biosciences) using primers for HSV-1 gB and mouse GAPDH. gB forward primer 5′- AACGCGACGCACATCAAG; gB reverse primer 5′- CTGGTACGCGATCAGAAAGC; GAPDH forward primer 5′- CATCACTGCCACCCAGAAGACTG; GAPDH reverse primer 5′- ATGCCAGTGAGCTTCCCGTTCAG. All primers were designed through IDT. BioRad CFX Opus 96 PCR system was used along with CFX Maestro Analysis Software: Cq values between 20 and 35 were considered positive. Ratios of gB to GAPDH were calculated using the Delta-Delta CT method.

For RT-qPCR, a soft palate containing NALT was collected directly into Trizol, homogenized using the GentleMACS M-tubes and program RNA_01 (Miltenyi Biotec) and RNA purified using the Qiagen RNeasy Kit according to the manufacturer’s instructions. Following elution with DEPC water, the samples were analyzed for yield and quality using an agarose gel. cDNA synthesis was performed using the BioRad iScript cDNA Synthesis Kit according to the manufacturer’s instructions and used for qPCR as described above substituting primers for HSV-1 LAT, ICP0, ICP4, and mouse NeuN and GAPDH. LAT forward primer 5′- CCTTATCTAAGGGCCGGCTG; LAT rev primer 5′- GGGACACATGCCTTCTTGGA; ICP4 forward primer 5′-CGGACCTGCTGTTTGAGAA; ICP4 rev primer 5′-CGGGACTCTTGCGCTTG; ICP0 forward primer 5’- TGATTGCCCGTCCAGATAAAG; ICP0 rev primer 5′-CAAGCTGGTGTACCTGATAGTG; NeuN forward primer 5’- CCATCTTCCTGTCCAAGTAGTC-3′; NeuN rev primer 5’- GGGACATTTCCGTGTCTGTAA-3′.

### Explant-induced reactivation

At 30  days post-infection (dpi), animals were humanely euthanized; the soft palate containing NALT (*n* = 4–5 animals, two separate experiments) was isolated as previously described (25). Soft palate containing NALT or isolated cell subtypes derived from NALT were incubated in MEM containing 2% charcoal-stripped FBS, antibiotics, and where denoted, 10  µM water-soluble DEX (Sigma; catalog no. D2915). Control samples explanted in MEM containing 2% charcoal-stripped FBS, antibiotics, and PBS were added at an equal volume to that of DEX. All cells were incubated in a CO2 incubator set at 37 °C and 5% CO2. For soft palate containing NALT, 500 µL aliquots were removed daily and used to perform plaque assays on Vero cell monolayers as previously described (20, 21). For individual cell subtypes, the cells were incubated with or without DEX for 24 h prior to plaquing on Vero monolayers.

### Fluorescence-activated cell sorting (FACS)

NALT were harvested from latently infected female mice (*n* = 10, two independent experiments) as described above. Single-cell suspensions were prepared as previously described (25). Briefly, soft palate containing NALT were pooled and homogenized against 100 µM mesh to break up cell aggregates. Serial centrifugation and washes were performed to remove debris. Remaining tissue fragments were subjected to mechanical dissociation using the gentleMACS C-tubes according to the manufacturer’s instructions (Miltenyi Biotec). Splenocytes were isolated and included as dual HSV-1 negative controls and positive controls for lymphocyte FACS gating strategies. Cells were suspended in MEM with 10% FBS and antibiotics with no phenol red (stain media) and trypan blue cell counting performed using the BioRad TC20 automated cell counter. Approximately 10^7^ cells were used for fluorescent staining with the denoted primary antibodies obtained from Novus biologics (Table 1) in the dark for 1 h at 4°C with gentle rocking. Washes and staining with a secondary antibody were performed as described for the primary antibody. Following secondary antibody staining, the cells were washed three times and suspended in 250 µL stain media with 3 µM propidium iodide (PI; Invitrogen) for 15 min prior to flow cytometric analysis and sorting.

Samples were analyzed on a BD FACSAria Special Order Research Product (Becton Dickinson, San Jose, CA) with assistance from the Immunopathology Core facility (Oklahoma Center for Respiratory and Infectious Diseases). Gating and downstream analysis were performed using BD FACSDiva Software. Gating proceeded from singlets (FSC vs. SSC) to PI live/dead to CD45^+^ lymphocytes to all downstream markers listed in Table 1. Gates were conservatively set to sort for purity over yield, and sorts were obtained collecting both stained cells and unsorted flowthrough separately. Figures were designed using BD FACSDiva Software and Floreida FCS analysis.

### Immunocytochemistry

Single-cell suspensions sorted TG neurons, or Neuro-2A cells (ATCC CCL-131), were cultured in 8-well chamber slides (NUNC) in MEM +5% FBS at 37°C, 5% CO2 for 24 h prior to staining. The cells were fixed with 4% paraformaldehyde in PBS for 15 min at room temperature, washed, and stained with antibodies against ICP0 (Abcam, ab6513, 1:500), ICP4 (Abcam, ab6514, 1:500), or NeuN (NovusBio, NBP1-92693SS, 1:500) for 1 h at room temperature. Cells were washed and stained with secondary antibody (Alexa-488, Invitrogen A-11029; 1:1000) or Alexa-594 (Invitrogen A-11005, 1:1000) for 1 h at room temperature, washed and stained with DAPI (4′,6-diamidino-2-phenylindole, dihydrochloride, ThermoFisher Scientific) for 10 min at room temperature prior to mounting of coverslips. Images were obtained with an Agilent BioTek Cytation 5 and Gen5 software (Fig. 6) or an Olympus BX microscope and CellSense Entry software (Fig. 10). Autoexposure was used unless indicated otherwise. The mean fluorescence intensity was calculated using ImageJ as previously described (51).

### Statistical analysis

All graphs and comparisons were performed using GraphPad Prism software (v10.2.2). *P* values less than 0.05 were considered significant for all calculations with specific tests and post-hoc analysis indicated in the respective figure legends.
